# Data in support of DPF2 regulates OCT4 protein level and nuclear distribution

**DOI:** 10.1016/j.dib.2015.10.010

**Published:** 2015-10-19

**Authors:** Chao Liu, Dijuan Zhang, Yuxian Shen, Xiaofang Tao, Lihua Liu, Yongwang Zhong, Shengyun Fang

**Affiliations:** a Department of Histology and Embryology, Institute of Stem Cell and Tissue Engineering, School of Basic Medical Sciences, Anhui Medical University, Hefei, Anhui 230032, China; b Center for Biomedical Engineering and Technology (BioMET), University of Maryland, Baltimore, MD 21201, USA; c School of Basic Medical Sciences, Institute of Biopharmaceuticals, Anhui Medical University, Hefei, Anhui 230032, China; d Institute of Clinical Pharmacology, Anhui Medical University, Hefei, Anhui 230032, China

## Abstract

DPF2, also named ubi-d4/requiem (REQU), interacts with a protein complex containing OCT4. This paper provides data in support of the research article entitled “DPF2 regulates OCT4 protein level and nuclear distribution”. The highlights include: (1) Denature-immunoprecipitation assay revealed ubiquitination of OCT4 in pluripotent H9 cells, which was enhancedby MG132, a proteasome inhibitor. (2) Well colocalization of ectopic OCT4 and FLAG-Ub was found in HeLa cells, which was also increased by MG132. (3) MG132 treatment decreased DPF2 cytoplasmic expression *in vivo*. These data give insights into how proteasome inhibition contributes to studying ubiquitnation of OCT4.

**Specifications** **table**

**Table:** 

Subject area	*Biology*
More specific subject area	*Molecular and cellular biology of stem cells, ubiquitination, localization.*
Type of data	*Figure, micrograph*
How data was acquired	*Western blot, fluorescence and immunofluorescence assay*
Data format	*Analyzed*
Experimental factors	*Ubiquitination of OCT4, distribution of DPF2 and OCT4 in the presence of MG132, a proteosome inhibitor.*
Experimental features	*Ubiquitination of OCT4 and distribution of DPF2 and OCT4 was investigated in H9 cells with or without the prescence of MG132. Colocalizationof ectopic OCT4 and FLAG-tag Ub was examined in HeLa cells by immunofluorescence assay.*
Data source location	*1.Anhui Medical University, Hefei, Anhui 230032 China2.University of Maryland, Baltimore, MD 21201 USA*
Data accessibility	*Data is with this article.*

## Value of the data

• Characterization of endogenous OCT4 ubiquitination was examined in H9 cells.
• Well colocalization of ectopic OCT4 and FLAG-Ub was identified in HeLa cells.
• MG132 treatment decreases cytoplasmic DPF2 in H9 cells and 293 cells.

## 1 Data

### 1.1 Ubiquitination of OCT4 in H9 cells

**Fig. 1:**
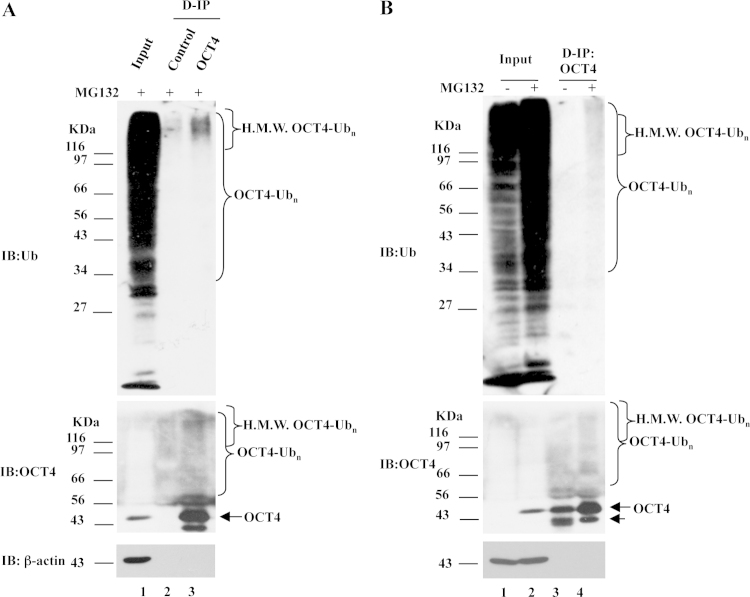
Characterization of OCT4 ubiquitination in H9 cells. (A) With the presence of MG132 for 6 h, H9 cells were subjected to IP assay with an anti-OCT4 antibody under denaturing conditions, followed by IB for indicated proteins. (B) With or without the presence of MG132 for 6 h, H9 cells were subjected to IP with an anti-OCT4 antibody under denaturing conditions followed by IB for indicated proteins. An arrow indicates the monomeric form of OCT4. An arrowhead indicates the lower band that was also recognized by the anti-OCT4 antibody. D-IP, IP under denaturing conditions. H.M.W. – high molecular weight.

We examined whether UPS plays role in regulating endogenous OCT4 protein level in H9 cells. The results showed that ubiquitinated OCT4 species, especially the high-molecular-weight ubiquitinated OCT4 species, were detected by both anti-OCT4 and anti-Ub antibodies (Fig. 1A, lane 3). Surprisingly, in cells treated with no MG132, OCT4 signals were not detectable in input (Fig. 1B, lane 1), although IP revealed the consistent presence of OCT4 (Fig. 1B, lane 3). Compared to the cells treated with no MG132 (Fig. 1B, lane 3), cells exposured to MG132 contain more ubiquitinated OCT4, especially the high-molecular-weight ubiquitinated OCT4 species (Fig. 1B, lane 4). Moreover, proteasome inhibition increased OCT4 protein level (Fig. 1B, lane 3 and lane 4, arrow), whereas level of the lower band below the monomeric form of OCT4, which was also recognized by anti-OCT4 antibody, decreased with the presence of MG132 (Fig. 1B, lane 3 and 4, arrowhead).

### 1.2 Well colocalization of OCT4 and FLAG-Ub in Hela cells

**Fig. 2:**
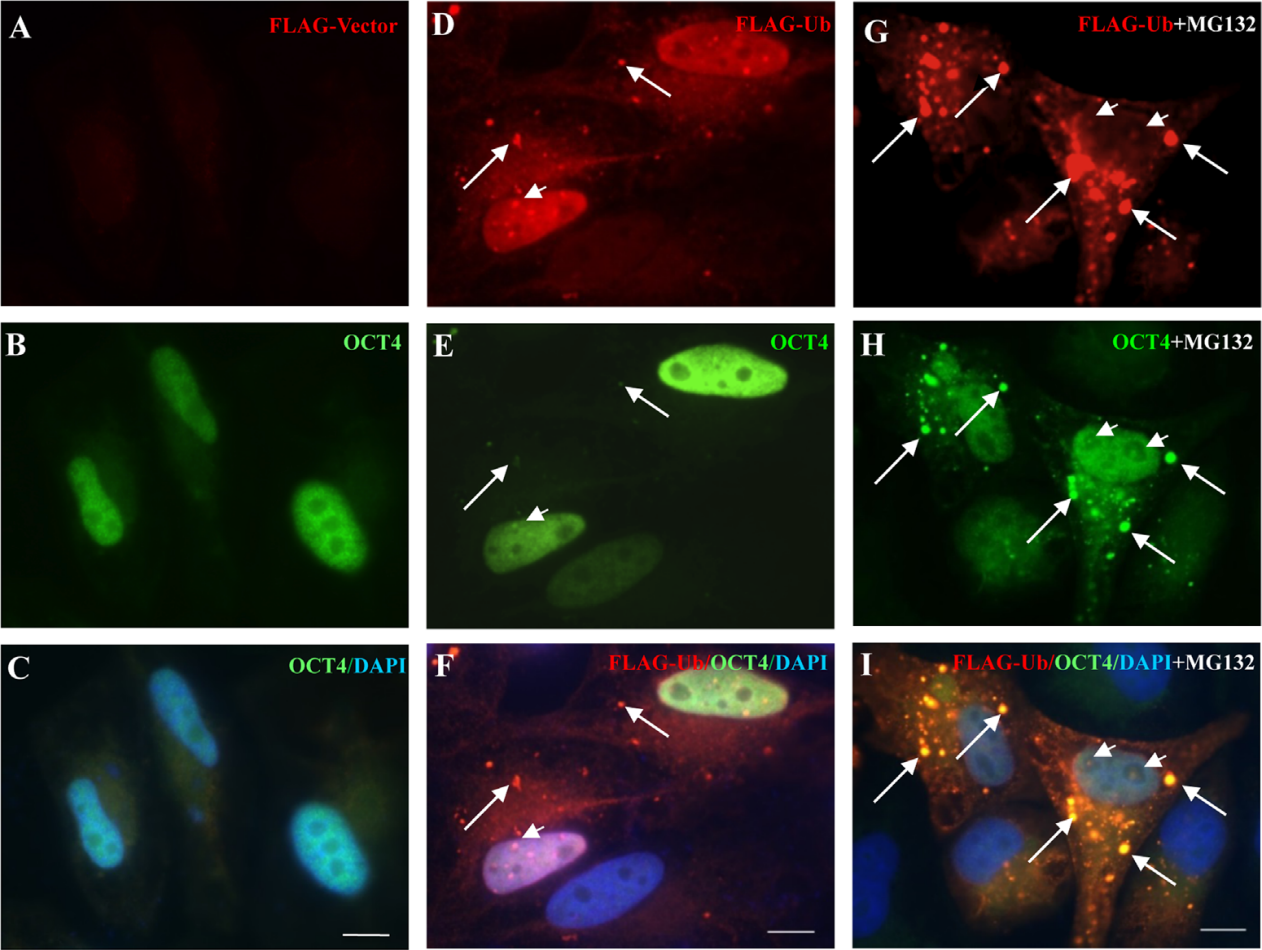
Coloclization of overexpressed OCT4 and FLAG-Ub in HeLa cells. Hela cells were cotransfected with plasmids encoding FLAG-Vector (A–C) and FLAG-DPF2 along (D–I) with OCT4. IF assay with indicated antibodies was performed 16 h after the transfection with (G–I) or without (D–F) the treatment of MG132 for 6 h. Bar=5 μm.

OCT4 ubiquitination has been addressed previously through biochemistry assay [1], [2], [3]. To investigate colocalization of OCT4 and Ub, we cotranfected HeLa cells with FLAG-Vector, and FLAG-Ub along with OCT4 plasmids. Immunofluorescence (IF) assay was performed with or without treatment of MG132. In cells coexpressed OCT4 and FLAG-Ub, well colocalization of Ub (Fig. 2D and F) and OCT4 (Fig. 2E and F) was identified in both cytoplasm (Fig. 2F, arrows) and nuclei (Fig. 2F, arrowheads). Treatment of MG132 promotes formation of aggregate-like structures positive for both ubiquitin and OCT4 (Fig. 2G–I).

### 1.3 MG132 treatment affects DPF2 subcellular localization

**Fig. 3:**
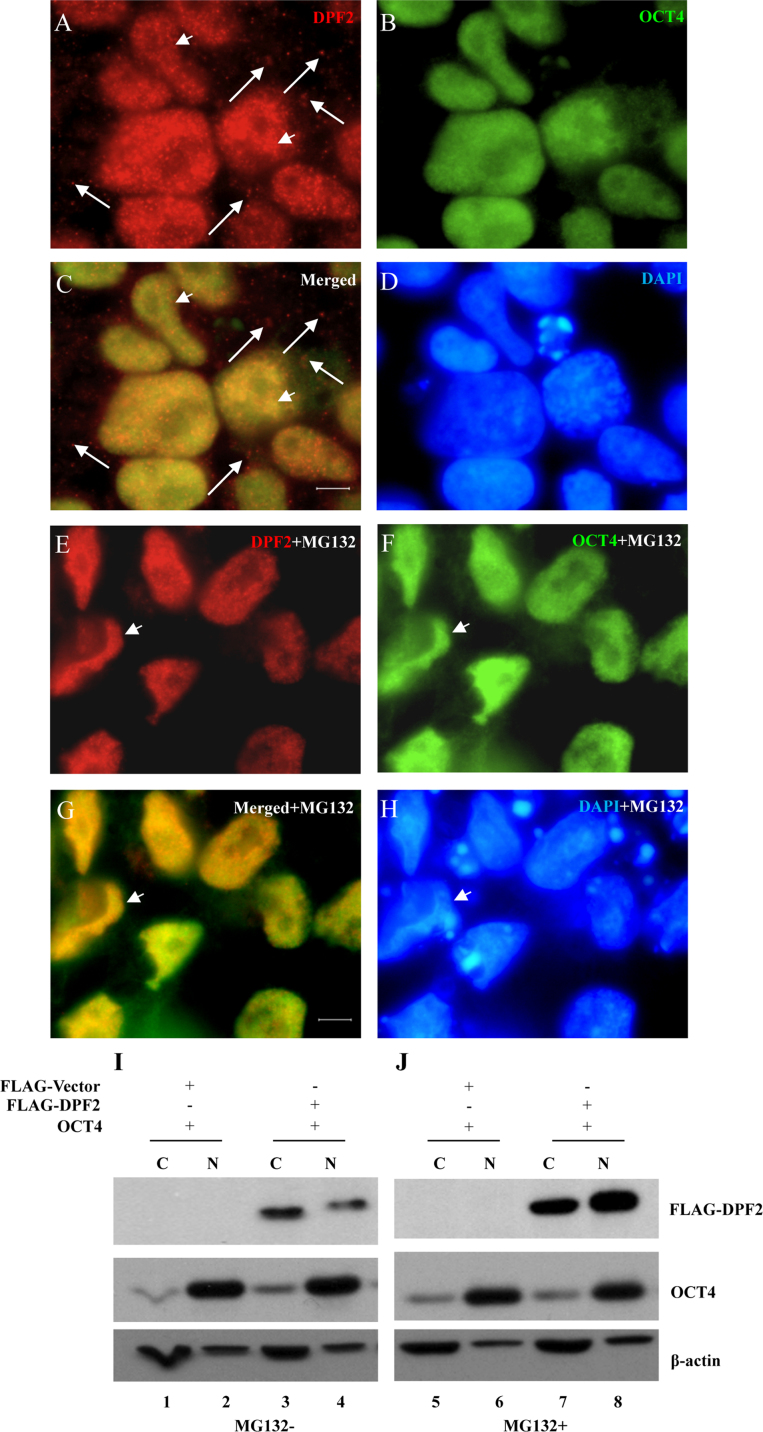
MG132 increases nuclear distribution of DPF2. (A–D) H9 cells were subjected to IF assay using anti-DPF2 (A and C, red) and anti-OCT4 (B and C, green) antibodies. Arrows indicate the cytoplasmic DPF2. E–H, H9 cells were subjected to IF assay using anti-DPF2 (E and H, red) and anti-OCT4 (F and H, green) antibodies in the presence of MG132. (I–J) 293 cells were cotransfected with FLAG-vector and FLAG-DPF2 (WT) along with OCT4 plasmids. After 16 h, the cells were treated with (lane 5–8) or without (lane 1–4) 20 μM MG132 for 6 h. Then the cells were subjected to cytoplasmic and nuclear fraction assay followed by IB using indicated antibodies.

DPF2, also named ubi-d4/requiem (REQU), interacts with OCT4 or a protein complex containing OCT4 *in vivo* [4], [5]. It contains double plant homeodomain (PHD) fingers, which functions in ubiquitination of target proteins [6], [7], [8], [9], [10], [11].Our recent work indicates DPF2 interact with and promotes ubiquitination of OCT4 (see Fig. 3 in [12]). Ubiquitination of proteins is also involved in regulating localization of themselves [13]. We therefore want to check intracellular localization of DPF2 and OCT4.H9 cells were subjected to IF assay with anti-DPF2 and anti-OCT4 antibodies with or without the presence of MG132. While DPF2 is expressed in both cytoplasm (Fig. 3A and C, red, arrows) and nuclei (Fig. 3A and C, red, arrowheads), OCT4 is expressed mainly in cell nuclei (Fig. 3B and C, green). Interestingly, MG132 treatment decreased the cytoplasmic expression of DPF2 (Fig. 3E and G, red) and induced accumulation of DPF2 in nuclei. OCT4 also colocalized with accumulatd DPF2 (Fig. 3F and G, red, arrowheads). The cytoplasmic and nuclear distribution of OCT4 seemed not changed by MG132 (Fig. 3). However, the more accumulated nuclear OCT4 expression is different from the diffusive OCT4 expression in H9 cells without treatment of MG132 (Fig. 3B and C *vs* F and G).

## 2 Experimental design, materials and methods

### 2.1 Cell culture

293 and HeLa cells werecultured as previously described [14], [15]. Human ESC line, H9, was maintained on Matrigel(BD Bioscience)-coated plates or coverslips in mTeSR medium (Stem Cell Technologies), as previously described [16], [17].

### 2.2 Examination of OCT4 ubiquitination in H9 cells by denature IP

H9 cells maintained on Matrigel in mTeSR medium were treated with 20 μM MG132 for 6 h. Cells were then subjected to denatured immunoprecipitation (IP) using control anti-goat antibodies and anti-OCT4 antibodies to disrupt proteins that may associate with OCT4, followed by IB for indicated proteins. Denatured IP was processed according to a previous protocol followed by IB assay as previously described [18].

### 2.3 IF assay of colocalization of ectopic OCT4 and FLAG-Ub in HeLa cells

HeLa cells were cotranfected by FLAG-Vector, and FLAG-Ub along with OCT4 plasmids. After 16 h, the cells were treated with or without MG132 for 6 h, then the cells were fixed and subjected to immunofluorescence assay using indicated antibodies.

### 2.4 Cytoplasmic and nuclear fraction assay

Cytoplasmic and nuclear fraction was performed as previous described [13]. The cells were harvested and suspended in 5 volumes of cold HB buffer (10 mM Tris, pH 7.9, 1.5 mM MgCl_2_, 10 mM KCl, protease inhibitor cocktail). After 15 min on ice, Triton X-100 was added to a final concentration of 0.2%. After vortexing for 5 s, the homogenate was spun for 10 min at 1000 g. The supernatant, containing the cytoplasmic fraction, was transferred to afresh tube, and the salt concentration was adjusted to 200 mM with 5 M NaCl. The crude nuclear pellet was suspended in RIPA-lysis buffer containing 1% Triton X-100 and 10% glycerol, and vortexed vigorously at 4 °C for 30 min. The homogenate was centrifuged for 15 min at 20,000 g. Nuclear and cytoplasmic factions were analyzed by IB as described.
